# Rapid Corneal Calcification Following Endothelial Keratoplasty: A Case Report

**DOI:** 10.7759/cureus.60956

**Published:** 2024-05-23

**Authors:** Atsuhiko Fukuto, Koichiro Shinji, Suzu Deie, Tai-ichiro Chikama

**Affiliations:** 1 Department of Ophthalmology and Visual Sciences, Graduate School of Biomedical and Health Sciences, Hiroshima University, Hiroshima, JPN; 2 Department of Ophthalmology, Hiroshima Prefectural Hospital, Hiroshima, JPN

**Keywords:** calcium phosphate, corneal calcification, persistent epithelial defect, dsaek, corneal opacity

## Abstract

Corneal calcification typically progresses slowly but can occasionally advance rapidly. This report details severe stromal calcification following repeat Descemet's stripping automated endothelial keratoplasty (DSAEK) in a 75-year-old patient with diabetes, hypertension, and prior ocular surgeries, including cataract surgery, intraocular lens extraction with suturing, and trabeculectomy. Persistent epithelial defects after the surgery led to rapid central stromal calcification within four weeks, significantly reducing visual acuity. Management included switching from betamethasone sodium phosphate to fluorometholone, facilitating complete epithelial recovery within two months. However, persistent stromal opacity necessitated a subsequent penetrating keratoplasty. Infrared absorption spectrophotometry identified calcium phosphate as the primary component of the calcification. This case highlights the importance of vigilant monitoring and proactive management of epithelial defects to prevent rapid calcification following endothelial keratoplasty.

## Introduction

Corneal calcification is a pathological condition characterized by the deposition of calcium salts within the cornea, leading to visual impairment. While typically a slow-progressing condition, it can occasionally advance rapidly, particularly in the presence of persistent epithelial defects (PEDs) [1]. PEDs disrupt the corneal barrier, allowing tear film components, such as calcium and phosphate, to infiltrate the stroma and precipitate as calcium salts.

Topical medications, particularly those containing phosphate, have been implicated in exacerbating corneal calcification. Continuous use of betamethasone sodium phosphate, for example, can react with tear calcium, leading to rapid stromal calcification, especially in eyes with compromised epithelial integrity [2].

This case report presents a 75-year-old male who developed severe stromal calcification following a repeat Descemet's stripping automated endothelial keratoplasty (DSAEK) in the presence of a PED. The report aims to provide insights into the potential mechanisms underlying this complication, the challenges in managing PEDs, and the importance of early intervention and appropriate therapeutic strategies to prevent rapid corneal calcification.

## Case presentation

**Figure 1:**
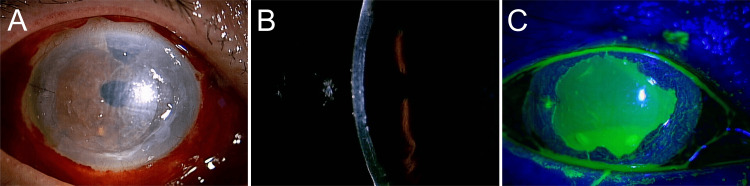
Slit-lamp microscopic image of the right eye, five days post DSAEK. (A) Diffuse illumination. (B) Slit-beam illumination reveals that the graft adhesion is satisfactory. (C) Fluorescein staining reveals extensive corneal epithelial defects. DSAEK: Descemet's stripping automated endothelial keratoplasty.

**Figure 2:**
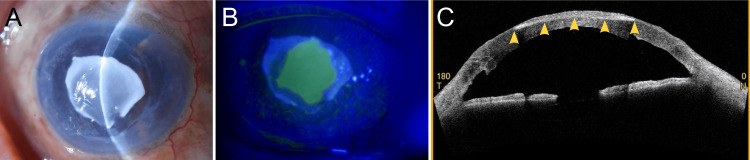
Anterior segment findings four weeks post DSAEK. (A) Calcification is observed in the central cornea. (B) Fluorescein staining reveals residual corneal epithelial defects. (C) The calcification of the corneal stroma appears as a hyperreflective area (arrowheads) on anterior segment optical coherence tomography. DSAEK: Descemet's stripping automated endothelial keratoplasty.

**Figure 3:**
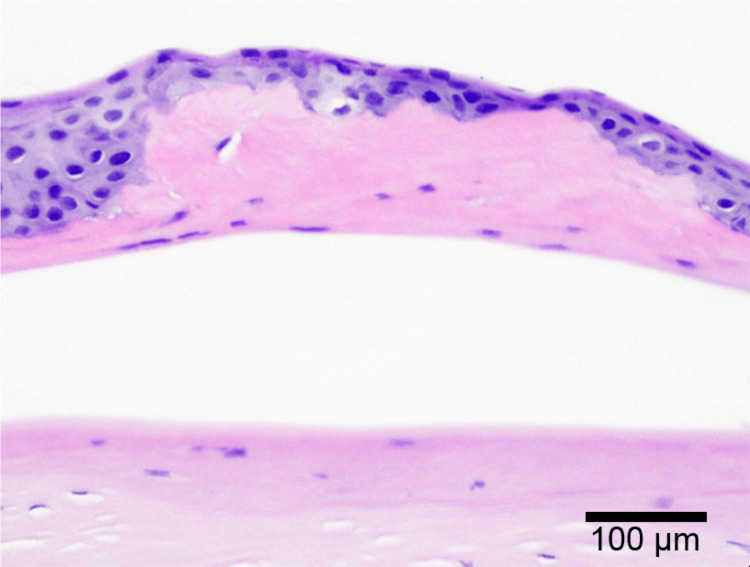
Histopathologic images of the excised cornea. Hematoxylin and eosin staining reveals a thick layer of calcification lesions immediately beneath the corneal epithelium (magnification, 100×).

**Figure 4:**
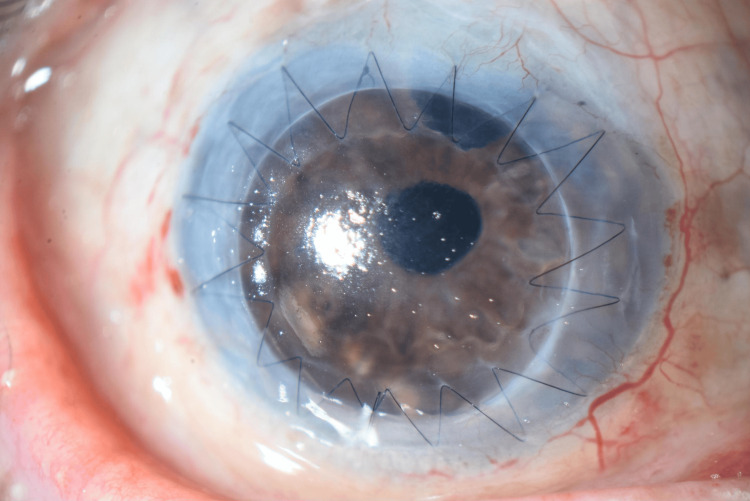
Anterior segment photograph of the right eye one year after penetrating keratoplasty.

A 75-year-old male presented with decreased visual acuity in the right eye for the past three months. The patient underwent standard cataract surgery (phacoemulsification and intraocular lens implantation, without intraoperative complications) in the right eye. Four years later, the patient experienced a dislocation of the intraocular lens (IOL), necessitating IOL extraction and suturing of a polymethyl methacrylate (PMMA) IOL. Another four years later, progressive visual field loss due to exfoliation glaucoma led to a trabeculectomy. Four years after that, the patient developed bullous keratopathy. Due to this condition, he was referred to Hiroshima University Hospital, where DSAEK was performed. During the first three years post DSAEK, the patient was under regular follow-up at our hospital with maintained graft clarity. However, subsequent regular follow-up was interrupted, and six years post DSAEK, the graft failed, necessitating a repeat DSAEK. Preoperative best-corrected visual acuity (BCVA) of the right eye was 20/100. Postoperative graft detachment was managed with five anterior chamber air injections within the first week. Repeated air injections led to extensive corneal epithelial loss (Figure 1). Despite achieving good graft adhesion, the extensive epithelial defect persisted post-discharge, resulting in central corneal calcification that extended into the deeper stroma by the fourth week (Figure 2), and decreased BCVA to 20/1000. Betamethasone sodium phosphate was suspected to contribute to calcification; hence, it was switched to fluorometholone four times daily. Complete epithelial healing was achieved two months postoperatively, but stromal opacity persisted, necessitating a penetrating keratoplasty (PK) at six months. Pathological examination of the excised cornea confirmed subepithelial calcification (Figure 3), with infrared absorption spectrophotometry identifying the deposits as calcium phosphate. One year post-PK, the transparency of the graft was maintained, and BCVA improved to 20/300 (Figure 4).

## Discussion

This case highlights rapid corneal stromal calcification following repeat DSAEK in a patient with a PED. Such rapid calcification is rare but significant, affecting visual prognosis and management.

Managing PED is challenging, especially in patients with a history of ocular surgeries. Our patient required repeated air injections after the second DSAEK, causing endothelial damage and delayed epithelial healing. Unlike some reports where soft contact lenses (SCL) were used to facilitate re-epithelialization and prevent calcification [3], we did not use SCLs initially due to observed healing trends. Early use of therapeutic lenses might have accelerated epithelial healing and prevented calcification. Factors contributing to PED include weak epithelial adhesion post-DSAEK and impaired healing in people with diabetes due to poor basal membrane adherence and reduced corneal sensation [4].

Rapid calcification can extend deeply into the stroma, differing from band keratopathy [1,5-7]. Continuous use of betamethasone sodium phosphate in the presence of an epithelial defect can cause rapid stromal calcification [2,8,9]. In this case, the use of betamethasone sodium phosphate eye drops likely contributed to the calcification process. Infrared absorption spectrophotometry was effective in identifying calcium phosphate in corneal deposits [10]. While calcium phosphate is the most common corneal calcium salt, rare cases related to *AGXT* gene mutations or exposure to oxalate-rich plant sap can result in calcium oxalate deposits [11,12].

This case underscores the importance of vigilant postoperative monitoring and early intervention in patients undergoing endothelial keratoplasty, particularly those with complex ocular histories. Early use of therapeutic contact lenses and careful management of topical medications may prevent rapid calcification in patients with persistent epithelial defects.

## Conclusions

PED following endothelial keratoplasty can lead to rapid corneal stromal calcification. In this case, multiple air injections for graft detachment management were likely a significant contributing factor. Preventing such calcification necessitates frequent examinations and aggressive treatment to promote re-epithelialization. Early intervention with therapeutic contact lenses and careful management of topical medications are crucial to mitigate the risk of rapid stromal calcification.
